# Molecular genetic characteristics of X-linked retinoschisis in Koreans

**Published:** 2009-04-23

**Authors:** So Yeon Kim, Hyun Soo Ko, Young Suk Yu, Jeong-Min Hwang, Jong Joo Lee, Sung Yeun Kim, Ji Yeon Kim, Moon-Woo Seong, Kyu Hyung Park, Sung Sup Park

**Affiliations:** 1 Department of Laboratory Medicine, Seoul National University Hospital, Seoul, Korea; 2 Clinical Research Institute, Seoul National University Hospital, Seoul, Korea; 3 Department of Ophthalmology, Seoul National University Hospital, Seoul, Korea; 4 Department of Ophthalmology, Seoul National University Bundang Hospital, Sungnam, Korea; 5 Department of Laboratory Medicine, National Cancer Center, Goyang, Korea

## Abstract

**Purpose:**

X-linked retinoschisis (XLRS) is a recessively inherited disorder that causes macular degeneration and resultant visual defect in young males. Many genetic studies had focused on the patients in Western countries. We characterized the mutational spectrum of the *RS1* gene in Korean patients with XLRS, and aimed to provide genetic information of XLRS in an Asian population.

**Methods:**

This study enrolled 17 unrelated probands and their mothers for molecular genetic evaluation. All exons and the flanking intronic regions of *RS1* were analyzed by direct sequencing. We performed gene dosage analysis by semiquantitative multiplex PCR to rule out the possibility of duplication in a patient without a sequence variation. We also tried RT–PCR analysis in a case with a putative splicing mutation.

**Results:**

Genetic tests revealed 16 Korean patients (94.1%) had *RS1* mutations. In one patient, neither sequence variation nor deletion or duplication in *RS1* was detected. One case with de novo mutation was confirmed by familial analysis. Identified were 14 causative mutations, three of which were novel: one missense mutation (c.227T>G, p.V76G) and two splice-site mutations (c.78+1G>T and c.78+5G>A). No obvious genotype-phenotype relationship was observed.

**Conclusions:**

A missense mutation was the predominant type, and common or founder mutations were not observed in the Korean patients in this study who had XLRS. This study provides molecular genetic characteristics about an Asian population previously unexplored. The genetic characteristics of Korean XLRS will be helpful for understanding the worldwide spectrum of *RS1* mutation.

## Introduction

X-linked retinoschisis (XLRS; OMIM 312700) is a recessively inherited bilateral vitreoretinal dystrophy that appears early in life, often in infancy [1]. The major symptom of affected males is reduced visual acuity [2]. Examination by ophthalmoscopy or optical coherence tomography usually reveals bilateral foveal schisis of variable degrees in retina. Other findings include peripheral retinoschisis, vitreous changes, and a characteristic selective reduction of the b wave of the electroretinogram [3]. Clinical presentation and disease severity can vary among patients, although the clinical diagnosis of XLRS is based on fundus abnormality, electrophysiological findings, and family histories that are consistent with X-linked inheritance [4].

Retinoschisin 1 (*RS1*), the only gene known to be associated with XLRS, is located in Xp22.2-p22.1. It spans approximately 15 kb, consists of six exons, and encodes a 24 kDa protein called retinoschisin [5,6]. This protein contains an evolutionarily conserved discoidin domain and exists as an octamer in which subunits are joined by intermolecular disulfide bonds [7]. The function of retinoschisin is unknown, but the observation that other members in the discoidin protein family are transmembrane or secreted adhesion proteins suggests that retinoschisin may also play a role in cell-to-cell adhesion [5,8].

Over 150 mutations in *RS1* have been reported in the Leiden open variation database for RS1. There are various types of mutations, but the majority of patients harbor missense mutations involving conserved amino acid residues within the discoidin domain.

The elucidation of mutation in various ethnicities is essential for understanding the causative gene and the pathogenesis of XLRS. In previous studies, the distributions of the *RS1* mutation were found to differ among ethnicities. Its worldwide prevalence ranges from 1:5,000 to 1:25,000 [9]. In Western countries, broad studies have been conducted. In Finland, XLRS is prevalent (>1:17,000), and approximately 95% of individuals of Finnish heritage have one of three founder mutations [10]. In other Western countries, studies involving large numbers of patients have shown heterogeneous mutation distribution without founder effect [11-14]. Reports describing the mutation spectrum in Asians have focused on relatively small numbers of probands. In Japanese patients, familial studies of three to 14 pedigrees [15-17] or studies of four to 11 unrelated patients [18-20] revealed that heterogeneous *RS1* aberrations were predominantly missense mutations. In Chinese patients, studies of 12 families [21] or five probands [22] also showed heterogeneous missense and small deletion mutations. However, in Koreans, only one XLRS family has been confirmed by molecular genetic diagnosis [23], and the prevalence and spectrum of *RS1* mutation were not known.

Therefore, a genetic study in Koreans as another Asian population will provide more information about worldwide *RS1* mutations. The current study aimed to identify the molecular genetic characteristics of XLRS in Koreans through more comprehensive analyses. We first performed exon duplication study in a case without sequence variation, and tried reverse-transcriptase PCR (RT–PCR) in a case with putative splicing mutation.

## Methods

### Patients

**Figure 1:**
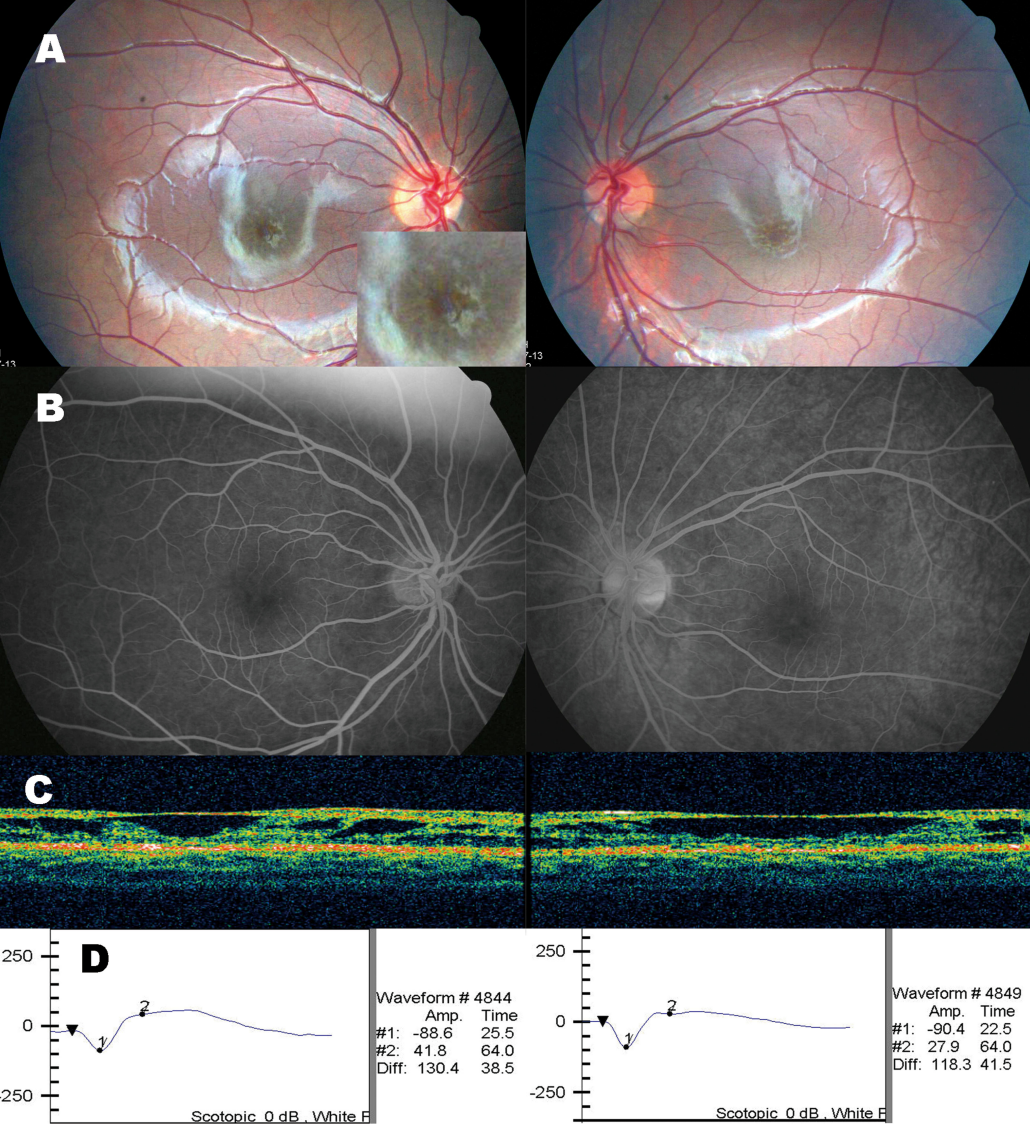
Ocular findings in a Korean XLRS patient (case 16). **A:** Fundus photograph of the both eyes showed typical stellate pattern of schisis cavities in the macula. The inset presents an image of the macula magnified twofold. **B:** Fluorescein angiogram showed no definite leakage from the cystic cavities. **C:** Optical coherence tomography showed the schisis in the nerve fiber layer. **D:** Electroretinogram showed markedly decreased amplitude of b-wave and relative preservation of a-wave, which are key features of XLRS.

This study included 17 unrelated Korean male patients and their mothers. Patients had visited outpatient clinics in Seoul National University Hospital or Seoul National University Bundang Hospital, with complaints of decreased visual acuity or strabismus. All patients were giving the diagnosis of XLRS by pediatric ophthalmologists (Y.S.Y. and J.M.H.), and an experienced retinal specialist (K.H.P.). Diagnosis was made primarily based on the presence of foveal schisis (stellate pattern of microcystic schisis cavities in the macula, Figure 1A), and peripheral schistic retinal changes were also evaluated with meticulous indirect ophthalmoscopic examination and slit lamp biomicroscope. If possible and affordable, other ancillary tests including electroretinography, optical coherence tomography, or fluorescein angiogram (for confirming no leakage from cystic macular lesions), were performed to confirm the diagnosis (Figure 1). Two of the patients had siblings with similar symptoms. Fifty-four female volunteers were recruited as normal controls after medical and ophthalmic examinations at the Health Promotion Clinic. The study protocol was approved by the institutional review board. All patients and their mothers gave their consent to participate in this study.

### Mutation analyses

#### DNA extraction and XY sex determination

Whole blood samples were obtained by venipuncture with anticoagulant EDTA tubes. They were stored in room temperature, and genomic DNA was extracted within 48 h using the PureGene DNA Isolation kit (Gentra Systems, Minneapolis, MN). Genetic sex determination of all participants was done by amplifying the zinc finger protein, X-linked gene (*ZFX*), and the zinc finger protein, Y-linked gene (*ZFY*). PCR products were then digested with the restriction enzyme HaeIII [24], and electrophoresed on 2.0% (w/v) agarose gels.

#### Direct sequencing of RS1

To detect sequence variations in all six exons and their flanking intronic regions, we designed primers (Table 1) and amplified *RS1* by PCR. The reaction was performed in a 20 μl volume containing 1X PCR buffer, 1.5 mM MgCl_2_, 0.2 mM dNTPs, 50 ng of DNA, 8 pmols of each primer, and 0.5 U of AmpliTaq Gold (Applied Biosystems, Foster City, CA). The cycling profiles were as follows: 95 °C for 10 min, 35 cycles of 30 s at 95 °C, 30 s at each annealing temperature, and 1 min at 72 °C. The final extension was at 72 °C for 7 min. PCR products were purified by ExoSAP-IT treatment (USB, Cleveland, OH) and bidirectionally sequenced on an ABI Prism 3730xl Genetic Analyzer (Applied Biosystems) using a BigDye Terminator Cycle Sequencing Ready Reaction Kit (Applied Biosystems). Obtained sequences were analyzed using Sequencher software ver 4.6 (Gene Codes Corporation, Ann Arbor, MI).

**Table 1:** Primers for the *RS1* mutation analysis.

**Gene**	**Usage**	**Exon**	**Primer (5′→3′)**
RS1	PCR and sequencing	1	F: GGTTAACTTGATGGGGCTCA
			R: CCCATCCTGTTTTCTGTTGG
		2	F: TTCTTCCAGAAGGGGTGTTG
			R: AAGCGATTCTCTTGCCTCAG
		3	F: TCAATTTGGCCATTGTAGCA
			R: GGAGAAAACCCGCATTAACA
		4	F: TGAACCGTTGAAGACACAGC
			R: AGTGCAGTGGTGTGATCTCG
		5	F: TTTCTTGGGAGGTGGAGATG
			R: GCAGATGATCCACTGTGCTG
		6	F: GTTCCAGATGTCCCAAGCAT
			R: TGCGAAATATAGCCCTGTCC
RS1	Gene dosage	1	F: GGGAAGATGTCACGCAAGAT
			R: AACTGGAAAGCCATCCACAC
		2	F: GCCACATTGGGATTATCGTC
			R: TGTTGGGATTACAGGCATGA
		3	F: AACCACAGTTGCCTTTGACC
			R: TGTTCCCAATGACTGTTCCA
		4	F: CAGTCACCTGGTGCTTGTTG
			R: CGAAGAATACCAGCCCACAT
		5	F: TTTCTTGGGAGGTGGAGATG
			R: TGTCCTGGAACTTGGAGAGC
		6	F: GTTCCAGATGTCCCAAGCAT
			R: GGTCCGAGTTGCCATAGAAG
HBB	Gene dosage	2	F: TTGGACCCAGAGGTTCTTTG
			R: GAGCCAGGCCATCACTAAAG
B2M	Gene dosage	2	F: CTCACGTCATCCAGCAGAGA
			R: AGTGGGGGTGAATTCAGTGT

Forward and reverse primers sequences were used to amplify the *RS1, HBB*, and *B2M* genes. Forward primers for gene dosage analysis were labeled with 6-FAM. Annealing temperature was 55 °C, with exception of *RS1* primers for amplification of exon 2 (60 °C), exon 4 (60 °C), and exon 5 (58 °C).

#### Gene dosage analysis of RS1

The gene dosage of *RS1* was assessed by semiquantitative multiplex PCR to detect duplication. Six exons of the *RS1* gene were amplified with the *HBB* gene and the *B2M* gene as endogenous references. Primer sequences are listed in Table 1. All of the forward primers were labeled with 6-FAM. After 18 cycles of PCR, the products were analyzed by ABI Prism 3130xl Genetic Analyzer (Applied Biosystems) with the GeneMapper ID 3.7 software. The peak height ratio of each exon of a patient was calculated by dividing the peak height of the *RS1* gene by that of the reference gene. Normalized dosage was determined by using the following equation:

$$\begin{array}{l} \text{Gene dosage}=\left[{\text{Peak}}_{\text{target}}\left(\text{patient}\right)/{\text{Peak}}_{\text{reference}}\left(\text{patient}\right)\right]/\\ \left[{\text{Peak}}_{\text{target}}\left(\text{control}\right)/{\text{Peak}}_{\text{reference}}\left(\text{control}\right)\right] \end{array}$$

the peak height ratio of each exon of a patient was divided by the ratio of a normal male control tested in parallel. Normalized values of *RS1* between 0.8 and 1.2 were considered as one copy of the exon, and between 1.8 and 2.2 as two copies. Healthy females with two X chromosomes used as two-copy controls of the *RS1* gene, in compared to males with one copy of *RS1*.

### Significance assessment of novel mutations

#### Allele frequency and in silico analysis

Normal frequency of novel sequence variation was determined in 108 normal X chromosomes by direct sequencing. We used Polymorphism Phenotyping (Polyphen) and Sorting Intolerant From Tolerant (SIFT) software programs to predict whether the novel missense mutation would affect protein function. Predictions were based on the position-specific independent counts (PSIC) score difference in Polyphen, and the tolerance index in SIFT [25,26]. The effect of a novel missense mutation was also assessed by the degree of interspecies amino acid conservation using the software Alamut. We also evaluated the effect of a putative splicing mutation by GeneSplicer, MaxEntScan, and SpliceSiteFinder-like using Alamut software.

#### RT–PCR trial in the case with a putative splicing mutation

RT–PCR was performed to confirm the effect of a novel putative splicing mutation, c.78+5G>A. Total RNA was isolated from leukocytes or lymphoblastoid cell lines of the patient using the RNeasy mini kit (Qiagen, Hilden, Germany). RT–PCR was performed using the Omniscript reverse transcriptase kit (Qiagen) and random hexamer or downstream primers. To detect minute amounts of aberrantly spliced RNA, we also used the whole transcriptome amplification kit (QuantiTect, Qiagen) with oligo(dT)_15_, random hexamer, or downstream primers. Primer sequences are shown in Table 2.

**Table 2:** Primers for RT–PCR of the *RS1* gene.

**Primer location**	**Range**	**Name**	**Sequence (5′->3′)**	**RNA amplification**	
				**Retina**	**Blood**
Exon 1 and 2	Exon 2–4	93F	GCCACATTGGGATTATCGTC	successful	successful
Exon 4		4aR	CGAAGAATACCAGCCCACAT		
Exon 1	Exon 1–6	36F	GGGAAGATGTCACGCAAGAT	successful	failed
Exon 6		6R	GGTCCGAGTTGCCATAGAAG		
Exon 1	Exon 1–6	48F	CGCAAGATAGAAGGCTTTTTG	successful	failed
Exon 6		6R	GGTCCGAGTTGCCATAGAAG		

Three primer pairs were designed to assess the effect of novel putative splicing mutation c.78+5G>A.

#### Paternity test for the de novo mutation

To confirm the de novo mutation, we performed paternity tests in the proband and his parents. This was done by using the AmpFlSTR profiler plus PCR amplification kit (Applied Biosystems). We analyzed ten genetic marker loci by using the software GeneMapper ID 3.7 (Applied Biosystems).

### Genotype-phenotype analysis

The possible relationship of genotype and phenotype was assessed between the type of mutation (missense versus intronic mutations) and clinical characteristics (age at symptom detection, coexistence of peripheral schisis, and comorbidity of vitreous hemorrhage) with the Fisher's exact test or the Mann–Whitney test. The possible effect of the changed amino acid (arginine versus others) was also analyzed in relation with clinical characteristics. Analyses were performed with SPSS for Windows version 12.0 (SPSS Inc., Chicago, IL). In each test, a *p*-value of less than 0.05 was considered statistically significant.

## Results

### Clinical characteristics of patients

The clinical characteristics of patients enrolled in this study are summarized in Table 3. The age of probands at the time of diagnosis varied from one month to 14 years. The mean age at diagnosis was 4.2±3.3 years. The initial presentation was poor visual acuity in older patients (5.8±3.0 years, 11 probands) or strabismus detected by their parents in younger patients (1.6±1.3 years, five probands). A one-month-old patient (case 13), born prematurely, had bilateral foveal schisis. All patients showed typical honeycomb appearance (foveal schisis) in the macula, and 82.4% of the probands also harbored peripheral schisis. Peripheral schisis was bilateral in 70.6% of the probands. In three patients with no schisis in periphery, two showed abnormal sheen in the inferior region of the retina (case 3 and case 9). Vitreous hemorrhage was the most common comorbidity (41.2%). Figure 1 shows representative clinical findings of a patient (case 16).

**Table 3:** Clinical characteristics of patients enrolled in this study.

**Case**	**RS1 mutation**	**Age at Dx**	**Symptom**	**VA/BCVA (R/L)**	**FS/PS**	**ERG**	**OCT**	**Ocular comorbidities**
1	p.R197H	14 Y	Poor VA, R	(0.02/0.3)/(0.04/0.4)	B/B, inferior	NT	NT	not found
2	p.R197C	4 Y	Poor VA, R	(0.02/0.2)/(0.02/0.2)	B/B, inferior	Reduced b wave	NT	R, VH
3	p.C142W	5 Y	Poor VA	(0.15/0.15)/(0.15/0.15)	B/not found	NT	NT	not found
4	c.78+1G>T	3 Y	Poor VA	(0.15/FC 50 cm)/(0.2/FC)	B/B, inferior	NT	NT	not found
5	p.L216P	5 Y	Familial history of poor VA	(0.2/0.15)/(0.3/0.2)	B/B, inferior	NT	NT	not found
6	p.V76G	8 M	Esodeviation since 5 M	(Mod/mod F&F)/(Mod/mod F&F)	B/B, inferior	Reduced b wave	NT	R, ISH
7	p.R102Q	1 Y	Esodeviation	(Mod/mod F&F)/(0.3/0.04)	B/B, inferior	NT	NT	L, VH
8	c.78+5G>A	3 Y	Poor VA since 11 M	(0.06/0.3)/(0.06/0.3)	B/B, inferior	NT	NT	B, VH
9	p.E72K	6 Y	Poor VA	(0.02/0.1)/(0.2/0.4)	B/not found	Reduced b wave	NT	not found
10	p.R213Q	3 Y	Esodeviation at 12 M	(0.1/HM)/(0.15/FC)	B/B, inferior	NT	NT	L, congenital cataract
11	p.R209C	6 Y	Visual disturbance	(0.3/0.3)/(0.4/0.4)	B/R, inferior	NT	NT	not found
12	p.R182C	3 M	Esodeviation	(Mod/mod F&F)/(0.08/0.04)	B/R, temporal & L, total	Reduced b wave	NT	B, VH&ISH
13	not found	1 M	Abnormal fundus findings with history of premature birth	(Poor/poor F&F)/NT	B/B	Flat b wave	NT	B, VH&SRH
14	p.R182C	6 Y	Poor VA, R	(0.2/0.3)/(0.2/0.3)	B/B, inferior	NT	NT	B, VH
15	p.E72Q	6 Y	Poor VA	(0.3/0.3)/(0.3/0.3)	B/L	Reduced b wave	B, FS	not found
16	p.E72K	6 Y	Poor VA	(0.15/0.15)/(0.3/0.2)	B/not found	Reduced b wave	B, FS	not found
17	p.R213W	3 Y	Esodeviation	(0.2/0.2)/(0.3/0.2)	B/B	Reduced b wave	B, FS&PS	not found

The clinical characteristics of Korean XLRS patients are summarized with their mutations. Abbreviations: diagnosis (Dx); visual acuity (VA); best corrected visual acuity (BCVA); foveal schisis (FS); peripheral schisis (PS); electroretinography (ERG); optical coherence tomography (OCT); years (Y); months (M); bilateral (B); right (R); left (L); finger counting (FC); fix and follow (F&F), hand movement (HM); moderate (mod); not tested (NT); vitreous hemorrhage (VH); intraschitic hemorrhage (ISH); subretinal hemorrhage (SRH).

### Molecular diagnosis and mutational spectrum

**Figure 2:**
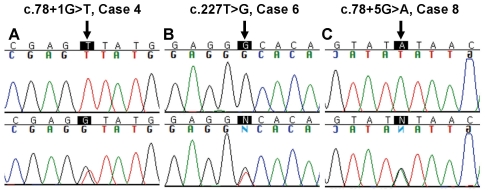
Sequences of three novel mutations identified in this study. **A:** c.78+1G>T was found in case 4. **B:** c.227T>G (p.V76G) was found in case 6. **C:** c.78+5G>A was found in case 8. The upper and lower panels reveal the *RS1* sequences of three probands and mothers, respectively. All three patients inherited the novel variants from their mothers. Positions of novel variants are indicated by arrows.

Among the 17 Korean patients, 16 males (94.1%) harbored causative *RS1* mutations and were given the diagnosis of XLRS based on genetic tests. In these 16 patients, 14 kinds of mutations were identified: 12 missense and two splice-site mutations. Three mutations were novel: one missense mutation (c.227T>G, p.V76G) and two splice-site mutations (c.78+1G>T and c.78+5G>A; Table 4 and Figure 2). Two intronic polymorphisms were also detected: c.184+35T>C and novel c.184+129T>G.

**Table 4:** Mutations and polymorphisms identified in the Korean XLRS patients

**Nucleotide change**	**Location**	**Effect of change**	**Case**	**Previous report**
c.78+1G>T	Intron 2	Splicing mutation	Case 4	Novel mutation
c.78+5G>A	Intron 2	Splicing mutation	Case 8	Novel mutation
c.184+35T>C	Intron 3	Polymorphism	Case 12, 14	Leiden open variation database for RS1
c.184+129T>G	Intron 3	Polymorphism	Case 12, 14	Novel variant
c.214G>A	Exon 4	p.E72K	Case 9, 16	[11]
c.214G>C	Exon 4	p.E72Q	Case 15	[36]
c.227T>G	Exon 4	p.V76G	Case 6	Novel missense mutation
c.305G>A	Exon 4	p.R102Q	Case 7	[11]
c.426T>G	Exon 5	p.C142W	Case 3	[37]
c.544C>T	Exon 6	p.R182C	Case 12, 14	[11]
c.589C>T	Exon 6	p.R197C	Case 2	[11]
c.590G>A	Exon 6	p.R197H	Case 1	[11]
c.625C>T	Exon 6	p.R209C	Case 11	[11]
c.637C>T	Exon 6	p.R213W	Case 17	[11]
c.638G>A	Exon 6	p.R213Q	Case 10	[15]
c.647T>C	Exon 6	p.L216P	Case 5	[11]

A de novo mutation was found in one patient. Case 3 was hemizygous for the p.C142W mutation, and neither parent had this mutation. We confirmed the parent-child relationships through paternity tests (data not shown).

**Figure 3:**
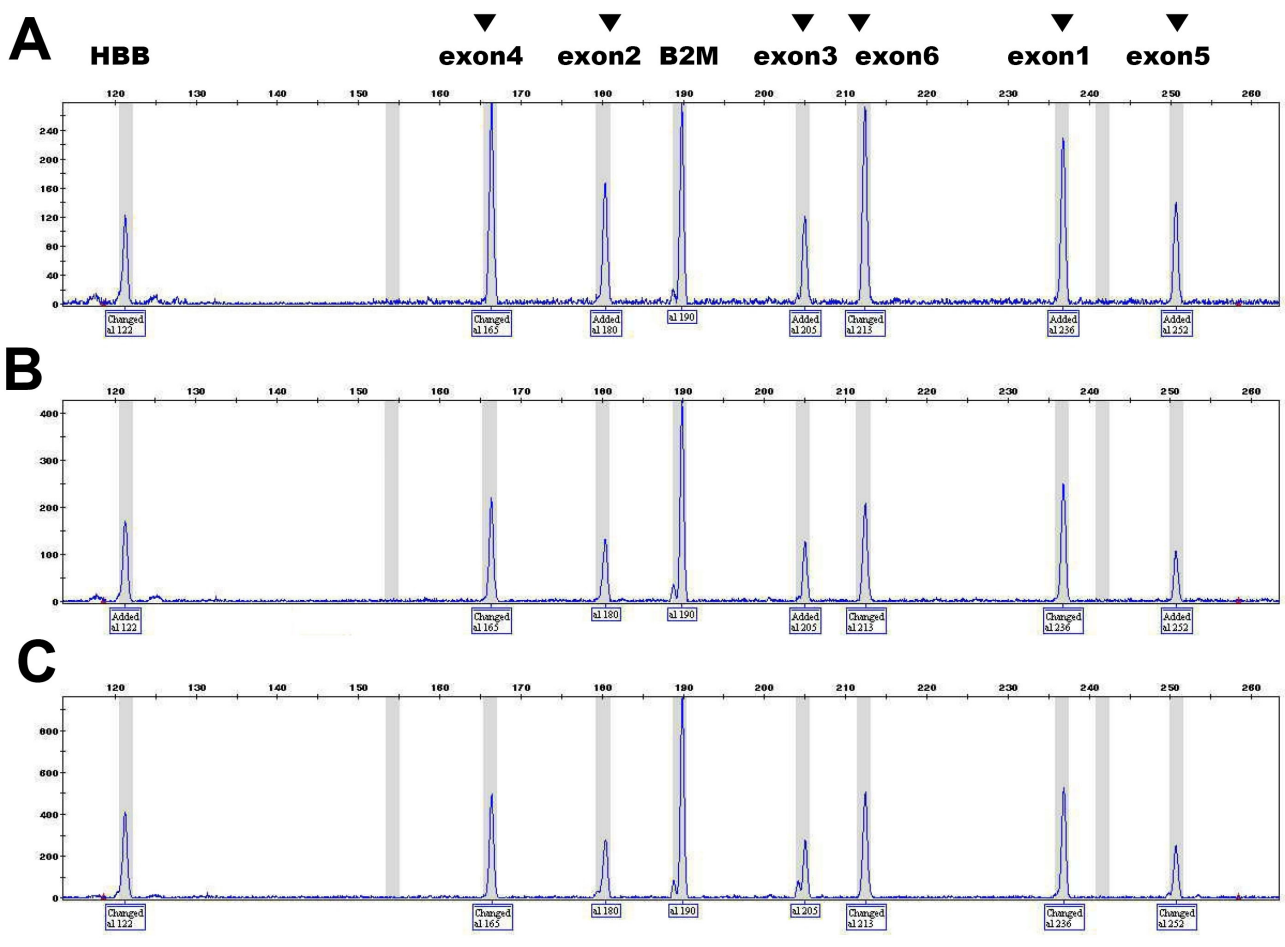
The representative chromatograms of gene dosage PCR. **A:** A normal female has two copies of the *RS1*. **B:** A normal male has one copy of the *RS1*. **C:** The chromatogram of case 13 showed one copy of the *RS1*. Each amplified products from six exons of *RS1* is indicated by the arrowhead above the fluorescent peak, and the size of each amplicon is shown below. The ratio calculated from the height of each peak is proportional to the targeted exon dosage, therefore duplication can be ruled out in the case 13. For example, the gene dosage of exon 4 was calculated as 1.0 in normal male, 2.09 in normal female, and 1.05 in case 13. Abbreviations: beta hemoglobin gene (*HBB*); beta-2-microglobulin gene (*B2M*).

Based on complete sequencing, one patient (case 13) with bilateral foveal schisis harbored no DNA sequence variation. By gene dosage analysis we revealed no *RS1* gene dosage alteration in this case (Figure 3), thus confirming that neither sequence variation nor duplication/deletion was the cause of his disease phenotype.

The most frequent cause of XLRS in Korean patients was the missense mutation (14/16; 87.5% of total mutation). All missense mutations identified in this study were located within the discoidin domain of retinoschisin. Contrary to previous reports of exon 4 as the site of highest mutation rate [3], exon 6 of the *RS1* gene was the most frequently involved region in Koreans (8/14). Two intronic mutations were located in intron 2. There was no predominant single mutation in Koreans. Each of two mutations, p.E72K and p.R182C, was found in two probands. The mutations in codon 72 (p.E72K and p.E72Q) were identified in three unrelated probands. The p.R182C mutation was linked to two polymorphisms, c.184+35T>C and c.184+129T>G, in two probands and their mothers.

### Significance assessment of novel mutations

The novel missense mutation, p.V76G, was expected to cause XLRS: The allele frequency was 0% in the normal control group. In the p.V76G mutation, the nonpolar amino acid valine was substituted by the smaller nonpolar glycine. This substitution was predicted to be harmful to protein function by in silico analysis. The tolerance index by SIFT was 0.00, which was below the cutoff (<0.05) score of a deleterious substitution. The PSIC score difference by Polyphen was 2.264, which was above the cutoff (>2.0) score of probably damaging. Codon 76 of retinoschisin is highly conserved in human and other species. Amino acid valine is at this position in *Pan troglodytes*, *Macaca mulatta, Rattus norvegicus, Mus musculus, Oryctolagus cuniculus, Canis familiaris, Bos taurus, Gallus gallus, Xenopus tropicalis, Tetraodon nigroviridis*, and *Danio rerio*. Isoleucine is only found in *Monodelphis domestica*.

One novel splice-site mutation, c.78+1G>T, was a definite causative mutation of XLRS. The allele frequency was 0% in normal controls, and the substitution within the invariant GT at the 5′ splice donor site was expected to block normal splicing. A different mutation involving the same nucleotide sequence was previously reported in human patients [27]. Another substitution in the same splice donor site of intron 2 activated a cryptic splice site in a murine model [28].

Another splice-site mutation, c.78+5G>A, was also predicted to be causative. Its allele frequency was 0% in normal controls. In the prediction software, c.78+5G>A introduced no alternative splice donor site. To determine the effect of this novel variant, we performed RT–PCR by using total RNA extracted from blood leukocytes and lymphoblastoid cell-lines. The *RS1* RNA was reliably detected in tissues of normal human retina with three different pairs of primers (Table 2). However, only a scarce amount of illegitimate *RS1* transcripts was detected in peripheral mononuclear cells using a pair of primers targeted between exon 1–2 junction and exon 4 (93F and 4aR), and two other primer pairs (36F and 6R, 48F and 6R) for amplifying between exon 1 and exon 6 did not work. There was no detectable *RS1* transcript in cDNA amplified using a whole transcriptome amplification kit. Even though the abnormal splicing product of c.78+5G>A could not be detected in peripheral cells, we predicted that this nucleotide change was pathogenic based on its normal frequency, the phenotype of the patient, and a genetic study of the mother.

### Genotype-phenotype correlation

Clinical phenotypes were not significantly different between patient group harboring missense mutations versus group harboring intronic mutations: age at symptom detection (Mann–Whitney test, p=0.267), presence of vitreous hemorrhage as an ocular comorbidity (Fisher's exact test, p=1.000), and coexistence of peripheral schisis (Fisher's exact test, p=1.000). Clinical manifestations were not significantly different between patient group harboring missense mutations involving arginine versus group harboring mutations not involving arginine: age at symptom detection (Mann–Whitney test, p=0.573) and presence of vitreous hemorrhage as an ocular comorbidity (Fisher's exact test, p=0.138). The relationship was also insignificant between the group harboring mutations involving arginine and the coexistence of peripheral schisis (Fisher's exact test, p=0.055). However, the possibility could not be completely excluded that more large studies would reveal the significance of arginine mutations.

## Discussion

In this study, we identified the mutation spectrum in Korean patients clinically diagnosed with XLRS. In 94.1% of probands, we detected known *RS1* gene mutation or novel genetic variation suspected as deleterious. No obvious genotype-phenotype association was observed in the Korean patients we studied.

Missense mutation in the discoidin domain is the major type of causative mutation in Korean patients. Any amino acid codon was not predominant for causative mutation in retinoschisin. In previous studies of different ethnicities, mutation spectrums were variable: p.E72K was the most common mutation in Western populations [11]. Among the Finnish patients, p.E72K mutation was in 70%, and p.G109R was in 19% [10]. However, no common mutation has been reported in Japanese or Chinese patients [15-21]. In the Korean population, a previously undefined ethnicity, mutation p.E72K was found in only two probands in this study. The dramatic mutational difference in Korean versus Finnish patients suggests that the mutation spectrum of Asian population may be different from those of some Western ethnicities. There was no apparent founder effect in Koreans, but the linkage between the p.R182C mutation and polymorphisms c.184+35T>C and c.184+129T>G in two patients and their mothers suggested the possibility of haplotypes of XLRS in Koreans.

Through this study, three novel mutations were found in the Korean population. Novel missense mutation p.V76G was located in the discoidin domain of retinoschisin, a well known region for causative mutations in XLRS. Two novel splice site mutations, c.78+1G>T and c.78+5G>A, were suspected to affect normal structure of protein. Each novel mutation was unique in each proband and his family. All three probands showed abnormalities indistinguishable from other Korean XLRS patients. These novel mutations widen the mutational spectrum of *RS1* and expect to help diagnosis of rare genetic disease XLRS in Asians or other ethnicities.

Previously, cysteine has been recognized as a residue frequently involved in *RS1* mutation. Even numbers of ten cysteine residues are present in retinoschisin, and the protein forms intermolecularly disulfide-bridged octamers, which are necessary for its biologic function [7]. Consequently, the sequence variant that changes the total cysteine number in retinischisin is considered a definite causative mutation. In this study, four sequence changes (4/12 missense mutation) involved cysteine residues, introducing loss or gain of a cysteine in retinoschisin. Interestingly, seven sequence changes involved arginine residues in 12 missense mutations. All of these sequence changes resulted in loss of an arginine. This mutation rate seemed higher than those reported in other large studies of XLRS [11,12] or those of other genetic diseases. Arginine is a well known mutated residue in proteins [29], and mutations at arginine residues account for almost 15% of the genetic disease mutations [30]. However, one previous report about XLRS also indicated frequent arginine involvement [13], and the role of some arginine residues in the structure of retinoschisin has been suggested in previous studies [31,32]. Therefore, the cause of apparently high mutability of arginine in this study and the effect of arginine mutation in XLRS need to be elucidated through further studies.

A proband with de novo mutation was found in this study. A de novo mutation is rare in XLRS. We confirmed de novo mutation through familial genetic tests. However, the possibility of germline mosaicism within the mother should be considered in subsequent genetic counseling and prenatal diagnosis.

One patient (case 13) in this study showed bilateral foveal and peripheral schisis indistinguishable from XLRS, but had no sequence variation within the *RS1* gene. It has been reported that no sequence alteration was found in approximately 10% of patients clinically compatible with XLRS [11,33]. In these patients, another cause of observed abnormalities should be considered such as other diseases than XLRS, or other genetic aberrations than sequence variation in the *RS1* gene. Clinically, the possibility could not be completely excluded, but least likely, that his comorbid condition such as premature birth had caused his schistic change, intra-schitic and vitreous hemorrhage, and electroretinographic findings. Genetically, deletion or duplication is one of the possible forms in the molecular genetic pathogenesis. Each exonal dosage test in the *RS1* is a novel diagnostic approach in cases with compatible phenotype of XLRS and with no sequence variation. Intragenic deletion was excluded in this case because of successful sequence amplification. Therefore we developed a gene dosage PCR, applied it to the case 13, and confirmed the absence of duplication within the *RS1*. Thus, in the clinically compatible, mutation-negative XLRS patients by direct sequencing, alternative approaches may be required: not only the gene dosage PCR successfully developed in this study but also future investigations such as analyses of the promoter regions, or search for other loci.

Existing in silico software can predict the consequence of a missense mutation in a protein [26]. Although not accurate enough, such programs are frequently used as an important tool. Novel missense mutation p.V76G was predicted by software to be harmful to protein structure and function. Two novel intronic variations, c.78+1G>T and c.78+5G>A, were also suggested as causative mutations of XLRS, given the phenotypes of the probands. The mutation in the invariant GT of a splice donor, c.78+1G>T, was a definite cause of XLRS. The novel change, c.78+5G>A, located slightly away from the exon-intron boundary, was suspected to affect splicing. The lack of the *RS1* gene transcript in blood cells has been reported since the 1990s. Transcripts of *RS1* have been detected in only a few organs outside retina such as human uterus [34] and pineal gland [35]. In this study, we detected the scarce amount of illegitimate transcription of *RS1* gene in blood cells. Although we found no working primer to detect abnormally spliced mRNA due to variant located intron 2, the effect of splicing variant could potentially be demonstrated experimentally under optimized conditions. Further work is required to explore the splicing variant in *RS1*.

In this study, no significant genotype-phenotype relationship was observed. Previous study about Caucasians showed no correlation between mutation type and severity of disease [12]. In Asian populations, no obvious association had been demonstrated in relatively small groups of Japanese or Chinese patients [15,20,22]. Phenotypic variability within the same genotype had been also noted in Japanese patients [16], and a few studies had implied the possibility of genotype-phenotype relationship: complication such as retinal detachment was apparently frequent in exonal deletion or p.R182C mutation in Japanese [17]. Phenotypes of XLRS were more severe in frameshift, splice site, or some missense mutations in Chinese [21]. The possibility of genotype-phenotype correlation and factors causing phenotypic variation are still needed more investigations.

The limitation of this study is only limited numbers of patients were performed electroretinogram and optical coherence tomography that might help to confirm the diagnosis. However, all the patients have bilateral stellate pattern of microcystic schisis cavities that was known as a pathognomonic sign of X-linked retinoschisis. 82% have additional peripheral retinal schisis which helps to differentiate with other diseases. Dominant cystoid macular edema usually did not accompany the peripheral retinal schisis. No patient showed pigmentary degeneration of retina and nyctalopia that was common in Goldmann-Favre syndrome and retinitis pigmentosa. No patient showed vitreous band or empty halo that might suggest the Sticker syndrome, Norrie disease and familial exudative vitreoretinopathy. No ocular inflammation including pars planitis was observed in the patients. Optic disc abnormalities such as congenital optic pit and morning glory syndrome were also not observed. Even though we did not completely rule out the other diseases mimicking X-linked retinoschisis, these clinical findings were quite robust evidence to differentiate X-linked retinoschisis from the other similar diseases.

The importance of clinical molecular genetics is increasing. Elucidation of the mutation spectrum and genotype-phenotype correlation contributes to the diagnosis and treatment of genetic ocular diseases. This study identified the genetic and clinical characteristics of XLRS in Koreans as an Asian ethnicity that had not been previously studied. Molecular techniques including gene dosage analysis were applied in this study. These will be helpful for diagnosis of XLRS and as confirmation when a clinical examination is difficult or the manifestation is uncommon.
